# Supplementary material for: From a Clustering of Adverse Symptoms after Colorectal Cancer Therapy to Chronic Fatigue and Low Ability to Work: A Cohort Study Analysis with 3 Months of Follow-Up
Source: Cancers (Basel). 2024 Jan 1;16(1):202. doi: 10.3390/cancers16010202 (PMC10778495; doi:10.3390/cancers16010202)
Supplement: Supplementary file 1 [file cancers-16-00202-s001.zip › cancers-2775456-supplementary.pdf]

Supplemental Material

From a Clustering of Adverse Symptoms After Colorectal Cancer Therapy to Chronic Fatigue and Ability to Work: A Cohort Study Analysis with 3 Months of Follow-up

Table of contents

Suppl. Table S1. Comparison of important baseline characteristics of participants who completed the 3-month follow-up, non-responding participants, and with late recruited participants who did not have a chance yet to participate in the 3-month follow-up 2

Suppl. Table S2. Interfactor correlation matrix of the identified 6 factors 3

Suppl. Table S3. Longitudinal associations of baseline covariates with the change in the fatigue score until 3-month follow-up 4

Suppl. Table S4. Longitudinal associations of symptoms at baseline with the change in the fatigue score until 3-month follow-up 5

Suppl. Table S5. Longitudinal associations of symptoms at with the change in the fatigue score until 3-month follow-up , subgroup analysis by sex 6

Suppl. Table S6. Longitudinal associations of symptoms at baseline with the change in the fatigue score until 3-month follow-up, subgroup analysis by age 7

Suppl. Table S7. Longitudinal associations of covariates at baseline with the change in the ability to work score until 3-month follow-up 8

Suppl. Table S8. Longitudinal associations of symptoms at baseline with the change in the ability to work score until 3-month follow-up 9

Suppl. Table S9. Longitudinal associations of symptoms at baseline with the change in the ability to work score until 3-month follow-up, subgroup analysis by sex 10

Suppl. Table S10. Longitudinal associations of symptoms at baseline the change in the ability to work score until 3-month follow-up, subgroup analysis by age 11

Suppl. Table S1. Comparison of important baseline characteristics of participants who completed the 3-month follow-up, non-responding participants, and with late recruited participants who did not have a chance yet to participate in the 3-month follow-up

**Baseline characteristics**	**Response at the 3-month follow up**				
	**Yes (N=279)**	**No (N=51)**		**Late recruitment (N=64)**	
	**Proportion (%)**	**Proportion (%)**	**P value [a]**	**Proportion (%)**	**P value [a]**
**Age (years)**					
< 65	164 (58.8)	27 (52.9)	0.437	31 (48.4)	0.132
≥ 65	115 (41.2)	24 (47.1)		33 (51.6)	
**Sex**					
Female	113 (40.5)	25 (49.0)	0.257	28 (43.7)	0.634
Male	166 (59.5)	26 (51.0)		36 (56.3)	
**Cancer stage**					
I	86 (31.4)	16 (32.0)	0.979	15 (29.4)	0.145
II	91 (33.2)	15 (30.0)		14 (27.5)	
III	69 (25.2)	14 (28.0)		16 (31.4)	
IV	14 (5.1)	3 (6.0)		6 (11.8)	
Unknown	14 (5.1)	2 (4.0)		0 (0)	

[a] Chi square test

Suppl. Table S2. Interfactor correlation matrix of the identified 6 factors

	Factor 1 "Fatigue"	Factor 2 "Gastro-intestinal symptoms"	Factor 3 "Pain"	Factor 4 "Psychosocial symptoms"	Factor 5 "Urinary symptoms"	Factor 6 "Chemotherapy side effects"
	**Pearson correlation coefficient r**					
Factor 1	N.A.					
Factor 2	0.34	N.A.				
Factor 3	0.54	0.48	N.A.			
Factor 4	0.32	0.23	0.27	N.A.		
Factor 5	0.56	0.16	0.33	0.32	N.A.	
Factor 6	0.53	0.22	0.32	0.18	0.42	N.A.

Abbreviation: N.A., not applicable

Suppl. Table S3. Longitudinal associations of baseline covariates with the change in the fatigue score until 3-month follow-up

Variable	β coefficient (95% CI)	p value
Age	0.06 (-0.06 ; 0.17)	0.335
Sex		
Female	1.00 Ref.	
Male	1.09 (-1.06 ; 3.24)	0.322
BMI (kg/m^2)		
> 25	1.00 Ref.	
25 – 30	-2.43 (-4.84 ; -0.02)	0.048
30 – 35	-1.13 (-4.31 ; 2.06)	0.488
> 35	-4.51 (-8.68 ; -0.35)	0.034
Stage		
1	1.00 Ref.	
2	0.61 (-1.98 ; 3.20)	0.645
3 and 4	-2.41 (-5.69 ; 0.86)	0.149
Unknown	1.26 (-3.63 ; 6.15)	0.614
Physical activity[a]		
No	1.00 Ref.	
Yes	0.52 (-1.60 ; 2.64)	0.630
Smoking		
Non smokers	1.00 Ref.	
Former smokers	0.10 (-2.13 ; 2.33)	0.932
Current smokers	-2.55 (-5.95 ; 0.85)	0.141
Number of comorbidities		
0	1.00 Ref.	
1	0.93 (-2.25 ; 4.10)	0.568
>=2	0.39 (-2.67 ; 3.45)	0.804
Months since surgery	0.38 (-1.91 ; 2.67)	0.745
Chemotherapy/radiation	4.71 (1.93 ; 7.48)	0.001
FACIT-F-FS at baseline	0.61 (0.52 ; 0.70)	< 0.001

[a] At least 150 min of moderate-intensity or 75 min of vigorous-intensity aerobic physical activity throughout the week

Suppl. Table S4. Longitudinal associations of symptoms at baseline with the change in the fatigue score until 3-month follow-up

Factor	Symptom	β Coefficient (95% CI)	P value
Fatigue	Dyspnea	-3.52 (-5.91; -1.12)	0.004
	Cognitive Fatigue	-2.93 (-5.84; -0.02)	0.048
	Depression	-2.43 (-5.07; 0.20)	0.070
	Emotional Fatigue	-1.95 (-4.84; 0.95)	0.187
	Appetite Loss	-1.84 (-4.87; 1.18)	0.233
	Social Sequelae	-1.84 (-4.90; 1.23)	0.240
	Interference with Daily Life	-1.05 (-4.03; 1.94)	0.492
	Ability to Work	0.14 (-2.25; 2.54)	0.906
Gastrointestinal symptoms	Embarrassment	-3.36 (-5.57; -1.15)	0.003
	Stool Frequency	-2.44 (-4.60; -0.28)	0.027
	Diarrhea	-2.32 (-4.46; -0.18)	0.033
	Sore Skin	-2.24 (-4.65; 0.18)	0.069
	Faecal Incontinence	-2.08 (-4.24; 0.09)	0.060
	Flatulence	0.13 (-2.22; 2.47)	0.915
	Blood or Mucus in Stool	0.70 (-1.78; 3.18)	0.579
Pain	Abdominal Pain	-5.11 (-7.86; -2.37)	< 0.001
	Dysuria	-3.98 (-7.01; -0.94)	0.010
	Buttock Pain	-2.56 (-5.08; -0.05)	0.046
	Pain	-1.92 (-4.16; 0.32)	0.092
	Bloating	-1.76 (-4.07; 0.56)	0.138
Psychosocial symptoms	Anxiety	-4.94 (-7.97; -1.91)	0.001
	Financial Difficulties	-3.37 (-5.60; -1.13)	0.003
	Psychosocial stress	-2.28 (-4.78; 0.23)	0.075
Urinary symptoms	Sleep Disturbance	-4.42 (-6.78; -2.05)	< 0.001
	Urinary Incontinence	-3.99 (-6.51; -1.47)	0.002
	Urinary Frequency	-0.93 (-3.10; 1.24)	0.402
Chemotherapy side effects	Constipation	-4.19 (-7.39; -0.99)	0.010
	Nausea or Vomiting	-3.70 (-6.31; -1.08)	0.006
	Taste	-1.53 (-3.96; 0.89)	0.215
	Hair Loss	-1.50 (-4.20; 1.19)	0.274

Note: Grey shade indicates p value <0.05

Suppl. Table S5. Longitudinal associations of symptoms at with the change in the fatigue score until 3-month follow-up , subgroup analysis by sex

Factor	Symptom	Females		Males	
		β coefficient (95% CI)	P value	β coefficient (95% CI)	P value
Fatigue	Cognitive Fatigue	-3.83 (-8.79; 1.13)	0.130	-1.57 (-5.43; 2.29)	0.425
	Ability to Work	-2.98 (-7.69; 1.73)	0.215	1.69 (-1.29; 4.68)	0.266
	Depression	-2.94 (-7.65; 1.78)	0.222	-2.33 (-5.51; 0.85)	0.151
	Interference with Daily Life	-2.83 (-8.50; 2.84)	0.328	-0.84 (-4.44; 2.77)	0.648
	Dyspnea	-2.09 (-6.71; 2.53)	0.376	-4.87 (-7.65; -2.09)	0.001
	Appetite Loss	-1.25 (-6.42; 3.93)	0.637	-3.31 (-7.65; 1.02)	0.134
	Emotional Fatigue	-0.39 (-5.66; 4.87)	0.884	-2.99 (-6.69; 0.71)	0.113
	Social Sequelae	2.09 (-3.26; 7.43)	0.444	-4.66 (-8.46; -0.87)	0.016
Gastrointestinal symptoms	Sore Skin	-5.21 (-9.33; -1.08)	0.013	-0.33 (-3.36; 2.71)	0.832
	Faecal Incontinence	-4.67 (-8.50; -0.83)	0.017	-0.00 (-2.76; 2.75)	0.998
	Stool Frequency	-3.56 (-7.49; 0.37)	0.076	-1.50 (-4.16; 1.16)	0.269
	Embarrassment	-3.53 (-7.47; 0.41)	0.079	-3.05 (-5.88; -0.23)	0.034
	Diarrhea	-3.27 (-7.40; 0.85)	0.120	-1.79 (-4.33; 0.76)	0.169
	Flatulence	-0.17 (-4.29; 3.96)	0.937	1.51 (-1.42; 4.45)	0.312
	Blood or Mucus in Stool	1.46 (-3.26; 6.18)	0.545	0.20 (-2.78; 3.19)	0.894
Pain	Dysuria	-9.87 (-15.72; -4.02)	0.001	-1.80 (-5.47; 1.87)	0.337
	Abdominal Pain	-5.88 (-10.40; -1.37)	0.011	-4.26 (-8.09; -0.42)	0.030
	Bloating	-5.14 (-8.86; -1.42)	0.007	1.49 (-1.58; 4.56)	0.342
	Pain	-4.89 (-9.13; -0.65)	0.024	0.03 (-2.69; 2.75)	0.984
	Buttock Pain	-4.62 (-8.71; -0.52)	0.027	-0.98 (-4.24; 2.28)	0.556
Psychosocial symptoms	Financial Difficulties	-6.40 (-10.42; -2.38)	0.002	-2.51 (-5.27; 0.25)	0.075
	Psychosocial stress	-5.57 (-10.11; -1.02)	0.016	-0.89 (-3.86; 2.08)	0.558
	Anxiety	-3.63 (-9.10; 1.85)	0.194	-5.00 (-8.75; -1.26)	0.009
Urinary symptoms	Sleep disturbances Disturbance	-4.04 (-8.31; 0.23)	0.063	-4.38 (-7.35; -1.40)	0.004
	Urinary Incontinence	-3.13 (-7.35; 1.08)	0.145	-5.10 (-8.39; -1.81)	0.002
	Urinary Frequency	-0.04 (-3.95; 3.87)	0.984	-1.61 (-4.24; 1.02)	0.230
Chemotherapy side effects	Hair Loss	-4.32 (-8.49; -0.15)	0.043	1.69 (-2.17; 5.56)	0.390
	Nausea or Vomiting	-4.24 (-8.65; 0.17)	0.060	-3.58 (-7.04; -0.13)	0.042
	Constipation	-3.22 (-8.68; 2.25)	0.248	-5.25 (-9.56; -0.94)	0.017
	Taste	-2.76 (-7.75; 2.23)	0.278	-1.75 (-4.53; 1.03)	0.218

Note: Grey shade indicates p value <0.05

Suppl. Table S6. Longitudinal associations of symptoms at baseline with the change in the fatigue score until 3-month follow-up, subgroup analysis by age

Factor	Symptom	Younger than 65 years		65 years and older	
		β coefficient (95% CI)	P value	β coefficient (95% CI)	P value
Fatigue	Dyspnea	-2.71 (-6.26; 0.84)	0.135	-4.49 (-8.10; -0.88)	0.015
	Cognitive Fatigue	-2.64 (-6.61; 1.32)	0.191	-2.25 (-6.75; 2.25)	0.327
	Emotional Fatigue	-2.54 (-6.56; 1.48)	0.215	-0.13 (-4.41; 4.14)	0.952
	Interference with Daily Life	-2.47 (-6.68; 1.73)	0.248	3.01 (-1.68; 7.71)	0.208
	Depression	-2.17 (-5.69; 1.35)	0.227	-3.82 (-8.00; 0.36)	0.073
	Social Sequelae	-1.65 (-6.11; 2.81)	0.468	-2.04 (-6.06; 1.99)	0.321
	Appetite Loss	-0.67 (-5.18; 3.85)	0.773	-4.20 (-8.35; -0.06)	0.047
	Ability to Work	-0.06 (-3.41; 3.30)	0.974	0.54 (-3.09; 4.18)	0.770
Gastrointestinal symptoms	Embarrassment	-4.58 (-7.61; -1.55)	0.003	-1.11 (-4.47; 2.25)	0.517
	Diarrhea	-3.76 (-6.85; -0.68)	0.017	0.14 (-3.09; 3.38)	0.931
	Sore Skin	-3.53 (-6.91; -0.14)	0.041	-0.71 (-4.27; 2.84)	0.694
	Stool Frequency	-2.81 (-5.79; 0.18)	0.065	-1.48 (-4.74; 1.77)	0.372
	Faecal Incontinence	-2.71 (-5.83; 0.41)	0.088	-0.25 (-3.45; 2.95)	0.879
	Blood or Mucus in Stool	2.54 (-1.02; 6.10)	0.161	-0.96 (-4.35; 2.43)	0.577
	Flatulence	-0.29 (-3.66; 3.09)	0.867	1.00 (-2.16; 4.16)	0.535
Pain	Abdominal Pain	-6.98 (-10.74; -3.22)	< 0.001	-2.86 (-7.09; 1.38)	0.186
	Dysuria	-5.43 (-9.75; -1.11)	0.014	-1.67 (-6.12; 2.78)	0.462
	Bloating	-3.49 (-6.76; -0.22)	0.036	0.84 (-2.48; 4.17)	0.618
	Pain	-3.07 (-6.31; 0.17)	0.063	0.02 (-3.16; 3.20)	0.989
	Buttock Pain	-2.42 (-5.86; 1.02)	0.168	-2.36 (-6.25; 1.53)	0.235
Psychosocial symptoms	Anxiety	-6.39 (-10.28; -2.49)	0.001	-1.91 (-6.93; 3.12)	0.457
	Financial Difficulties	-4.08 (-7.21; -0.94)	0.011	-2.38 (-5.76; 1.00)	0.167
	Psychosocial stress	-1.88 (-5.35; 1.60)	0.290	-1.70 (-5.44; 2.04)	0.372
Urinary symptoms	Urinary Incontinence	-5.14 (-8.90; -1.37)	0.007	-1.40 (-4.84; 2.04)	0.426
	Sleep disturbances	-3.89 (-7.21; -0.57)	0.022	-4.50 (-7.93; -1.08)	0.010
	Urinary Frequency	-0.31 (-3.37; 2.74)	0.840	-0.42 (-3.76; 2.91)	0.803
Chemotherapy side effects	Constipation	-5.30 (-9.51; -1.09)	0.014	-1.31 (-6.69; 4.07)	0.633
	Nausea or Vomiting	-3.78 (-7.40; -0.17)	0.040	-3.69 (-7.50; 0.12)	0.058
	Hair Loss	-1.39 (-5.04; 2.26)	0.456	-2.43 (-6.59; 1.74)	0.253
	Taste	-1.21 (-4.79; 2.37)	0.508	-1.08 (-4.45; 2.29)	0.531

Note: Grey shade indicates p value <0.05

Suppl. Table S7. Longitudinal associations of covariates at baseline with the change in the ability to work score until 3-month follow-up

**Variable**	**β coefficient (95% CI)**	**p value**
Age	0.00 (-0.02; 0.01)	0.706
Sex		
Female	1.00 Ref.	
Male	0.06 (-0.21; 0.32)	0.678
BMI (kg/m^2)		
> 25	1.00 Ref.	
25 – 30	0.09 (-0.22; 0.39)	0.580
30 – 35	0.07 (-0.33; 0.46)	0.742
> 35	-0.37 (-0.90; 0.15)	0.161
Stage		
1	1.00 Ref.	
2	-0.07 (-0.39; 0.26)	0.688
3 and 4	-0.27 (-0.68; 0.14)	0.200
Unknown	-0.22 (-0.84; 0.39)	0.479
Physical activity[a]		
No	1.00 Ref.	
Yes	-0.00 (-0.27; 0.27)	0.982
Smoking		
Non smokers	1.00 Ref.	
Former smokers	-0.13 (-0.41; 0.15)	0.367
Current smokers	-0.32 (-0.75; 0.11)	0.146
Number of comorbidities		
0	1.00 Ref.	
1	0.12 (-0.28; 0.53)	0.544
>=2	-0.15 (-0.54; 0.23)	0.434
Months since surgery	-0.23 (-0.53; 0.06)	0.119
Chemotherapy/radiation	0.39 (0.04; 0.74)	0.030
FACIT-F-FWB Ability to Work item at baseline	0.53 (0.42; 0.63)	< 0.001

[a] At least 150 min of moderate-intensity or 75 min of vigorous-intensity aerobic physical activity throughout the week

Suppl. Table S8. Longitudinal associations of symptoms at baseline with the change in the ability to work score until 3-month follow-up

Factor	Symptom	β Coefficient (95% CI)	P value
Fatigue	Appetite Loss	-0.66 (-1.00; -0.31)	< 0.001
	Emotional Fatigue	-0.66 (-0.95; -0.37)	< 0.001
	Fatigue (FACIT-F)	-0.63 (-0.92; -0.34)	< 0.001
	Depression	-0.50 (-0.79; -0.21)	0.001
	Cognitive Fatigue	-0.50 (-0.83; -0.17)	0.003
	Interference with Daily Life	-0.48 (-0.76; -0.21)	0.001
	Social Sequelae	-0.44 (-0.80; -0.09)	0.015
	Dyspnea	-0.40 (-0.70; -0.11)	0.007
Gastrointestinal symptoms	Embarrassment	-0.68 (-0.95; -0.41)	< 0.001
	Faecal Incontinence	-0.37 (-0.64; -0.11)	0.006
	Sore Skin	-0.34 (-0.65; -0.03)	0.030
	Diarrhea	-0.34 (-0.59; -0.08)	0.011
	Stool Frequency	-0.12 (-0.39; 0.16)	0.401
	Flatulence	-0.04 (-0.32; 0.24)	0.787
	Blood or Mucus in Stool	0.08 (-0.23; 0.40)	0.596
Pain	Abdominal Pain	-0.64 (-0.99; -0.29)	< 0.001
	Pain	-0.56 (-0.83; -0.29)	< 0.001
	Dysuria	-0.54 (-0.92; -0.16)	0.005
	Buttock Pain	-0.54 (-0.84; -0.23)	0.001
	Bloating	-0.34 (-0.63; -0.06)	0.019
Psychosocial symptoms	Anxiety	-0.73 (-1.07; -0.39)	< 0.001
	Psychosocial stress	-0.51 (-0.78; -0.24)	< 0.001
	Financial Difficulties	-0.39 (-0.67; -0.11)	0.006
Urinary symptoms	Sleep Disturbance	-0.63 (-0.90; -0.36)	< 0.001
	Urinary Incontinence	-0.23 (-0.55; 0.08)	0.148
	Urinary Frequency	-0.09 (-0.37; 0.18)	0.502
Chemotherapy side effects	Nausea or Vomiting	-0.83 (-1.12; -0.53)	< 0.001
	Taste	-0.49 (-0.79; -0.20)	0.001
	Constipation	-0.45 (-0.84; -0.05)	0.027
	Hair Loss	-0.16 (-0.50; 0.18)	0.368

Note: Grey shade indicates p value <0.05

Suppl. Table S9. Longitudinal associations of symptoms at baseline with the change in the ability to work score until 3-month follow-up, subgroup analysis by sex

Factor	Symptom	Female		Male	
		**β coefficient (95% CI)**	**P value**	**β coefficient (95% CI)**	**P value**
Fatigue	Cognitive Fatigue	-0.58 (-1.09; -0.08)	0.022	-0.35 (-0.84; 0.14)	0.157
	Depression	-0.49 (-0.95; -0.04)	0.035	-0.55 (-0.95; -0.15)	0.007
	Emotional Fatigue	-0.40 (-0.88; 0.08)	0.103	-0.82 (-1.22; -0.42)	< 0.001
	Interference with Daily Life	-0.40 (-0.88; 0.07)	0.094	-0.54 (-0.91; -0.17)	0.004
	Appetite Loss	-0.35 (-0.93; 0.23)	0.239	-0.98 (-1.48; -0.48)	< 0.001
	Fatigue (FACIT-F)	-0.35 (-0.88; 0.19)	0.204	-0.82 (-1.18; -0.47)	< 0.001
	Social Sequelae	-0.25 (-0.82; 0.32)	0.396	-0.68 (-1.17; -0.20)	0.006
	Dyspnea	-0.16 (-0.69; 0.36)	0.544	-0.61 (-0.98; -0.23)	0.001
Gastrointestinal symptoms	Embarrassment	-0.65 (-1.10; -0.21)	0.004	-0.68 (-1.05; -0.31)	< 0.001
	Sore Skin	-0.53 (-1.02; -0.04)	0.033	-0.25 (-0.67; 0.17)	0.247
	Faecal Incontinence	-0.43 (-0.87; 0.02)	0.061	-0.33 (-0.70; 0.04)	0.081
	Blood or Mucus in Stool	0.36 (-0.18; 0.89)	0.191	-0.06 (-0.47; 0.35)	0.783
	Diarrhea	-0.15 (-0.62; 0.33)	0.541	-0.43 (-0.76; -0.10)	0.010
	Stool Frequency	-0.08 (-0.54; 0.38)	0.724	-0.15 (-0.51; 0.21)	0.419
	Flatulence	0.04 (-0.41; 0.49)	0.873	-0.04 (-0.43; 0.36)	0.858
Pain	Dysuria	-0.92 (-1.60; -0.24)	0.008	-0.40 (-0.89; 0.10)	0.115
	Abdominal Pain	-0.79 (-1.30; -0.28)	0.003	-0.47 (-0.99; 0.05)	0.077
	Pain	-0.77 (-1.25; -0.29)	0.002	-0.45 (-0.81; -0.10)	0.012
	Buttock Pain	-0.65 (-1.11; -0.19)	0.005	-0.44 (-0.89; 0.01)	0.055
	Bloating	-0.41 (-0.85; 0.03)	0.070	-0.24 (-0.66; 0.17)	0.252
Psychosocial symptoms	Anxiety	-0.64 (-1.19; -0.10)	0.021	-0.71 (-1.18; -0.24)	0.003
	Financial Difficulties	-0.53 (-1.01; -0.05)	0.032	-0.38 (-0.75; -0.02)	0.041
	Psychosocial stress	-0.45 (-0.92; 0.02)	0.063	-0.61 (-0.95; -0.26)	0.001
Urinary symptoms	Sleep disturbances	-0.60 (-1.05; -0.14)	0.011	-0.61 (-0.98; -0.25)	0.001
	Urinary Incontinence	-0.24 (-0.72; 0.24)	0.330	-0.28 (-0.73; 0.18)	0.233
	Urinary Frequency	-0.06 (-0.51; 0.40)	0.810	-0.15 (-0.51; 0.22)	0.424
Chemotherapy side effects	Nausea or Vomiting	-0.95 (-1.42; -0.49)	< 0.001	-0.85 (-1.27; -0.43)	< 0.001
	Taste	-0.39 (-0.97; 0.18)	0.182	-0.60 (-0.96; -0.24)	0.001
	Constipation	-0.33 (-0.94; 0.29)	0.299	-0.52 (-1.09; 0.06)	0.078
	Hair Loss	-0.17 (-0.66; 0.33)	0.507	-0.06 (-0.59; 0.46)	0.816

Note: Grey shade indicates p value <0.05

Suppl. Table S10. Longitudinal associations of symptoms at baseline the change in the ability to work score until 3-month follow-up, subgroup analysis by age

Factor	Symptom	Younger than 65 years		65 years and older	
		β coefficient (95% CI)	P value	β coefficient (95% CI)	P value
Fatigue	Appetite Loss	-0.76 (-1.28; -0.25)	0.004	-0.77 (-1.26; -0.28)	0.002
	Fatigue (FACIT-F)	-0.72 (-1.14; -0.30)	0.001	-0.64 (-1.06; -0.23)	0.002
	Social Sequelae	-0.67 (-1.18; -0.17)	0.008	-0.15 (-0.67; 0.37)	0.566
	Emotional Fatigue	-0.57 (-0.97; -0.17)	0.005	-0.90 (-1.35; -0.45)	< 0.001
	Interference with Daily Life	-0.53 (-0.93; -0.14)	0.008	-0.44 (-0.88; -0.01)	0.046
	Cognitive Fatigue	-0.45 (-0.90; -0.01)	0.047	-0.61 (-1.16; -0.07)	0.027
	Depression	-0.41 (-0.80; -0.01)	0.044	-0.67 (-1.14; -0.20)	0.005
	Dyspnea	-0.30 (-0.74; 0.13)	0.171	-0.62 (-1.08; -0.17)	0.007
Gastrointestinal symptoms	Embarrassment	-0.81 (-1.17; -0.44)	< 0.001	-0.49 (-0.93; -0.05)	0.028
	Faecal Incontinence	-0.45 (-0.84; -0.07)	0.022	-0.31 (-0.73; 0.11)	0.145
	Diarrhea	-0.40 (-0.78; -0.02)	0.038	-0.34 (-0.75; 0.07)	0.101
	Blood or Mucus in Stool	0.35 (-0.09; 0.79)	0.120	-0.25 (-0.71; 0.21)	0.281
	Sore Skin	-0.33 (-0.76; 0.10)	0.134	-0.43 (-0.91; 0.05)	0.078
	Flatulence	-0.14 (-0.53; 0.26)	0.492	0.04 (-0.38; 0.47)	0.839
	Stool Frequency	-0.03 (-0.41; 0.35)	0.858	-0.28 (-0.71; 0.16)	0.215
Pain	Abdominal Pain	-0.73 (-1.20; -0.26)	0.002	-0.67 (-1.22; -0.11)	0.018
	Dysuria	-0.66 (-1.21; -0.11)	0.018	-0.34 (-0.95; 0.26)	0.266
	Pain	-0.61 (-1.01; -0.20)	0.003	-0.54 (-0.93; -0.15)	0.007
	Buttock Pain	-0.48 (-0.90; -0.07)	0.023	-0.64 (-1.16; -0.12)	0.015
	Bloating	-0.43 (-0.82; -0.03)	0.035	-0.15 (-0.61; 0.30)	0.509
Psychosocial symptoms	Anxiety	-0.74 (-1.19; -0.29)	0.001	-0.75 (-1.31; -0.20)	0.008
	Psychosocial stress	-0.44 (-0.83; -0.05)	0.026	-0.66 (-1.06; -0.27)	0.001
	Financial Difficulties	-0.41 (-0.80; -0.02)	0.038	-0.27 (-0.72; 0.18)	0.242
Urinary symptoms	Sleep disturbances	-0.64 (-1.04; -0.25)	0.001	-0.61 (-1.01; -0.21)	0.003
	Urinary Incontinence	-0.31 (-0.79; 0.17)	0.205	-0.18 (-0.63; 0.27)	0.435
	Urinary Frequency	-0.18 (-0.56; 0.20)	0.360	0.07 (-0.39; 0.54)	0.757
Chemotherapy side effects	Nausea or Vomiting	-0.88 (-1.28; -0.47)	< 0.001	-0.82 (-1.28; -0.36)	< 0.001
	Constipation	-0.56 (-1.07; -0.05)	0.032	-0.24 (-0.94; 0.47)	0.512
	Taste	-0.45 (-0.88; -0.02)	0.039	-0.56 (-1.00; -0.11)	0.015
	Hair Loss	-0.14 (-0.59; 0.32)	0.553	-0.24 (-0.82; 0.35)	0.427

Note: Grey shade indicates p value <0.05
